# Fever‐Induced Heat Shock Protein‐70 Regulates Macrophage IL‐1β and IL‐10 Secretion During *Mycobacterium tuberculosis* Infection

**DOI:** 10.1002/eji.202551963

**Published:** 2025-07-16

**Authors:** Deborah L. W. Chong, Sajeel A. Shah, Julia Kutschenreuter, Ramla Cusman, Meena Murugananden Pillai, Daniela E. Kirwan, Robert H. Gilman, Jon S. Friedland

**Affiliations:** ^1^ Institute for Infection and Immunity, City St George's University of London London UK; ^2^ Department of International Health Johns Hopkins University Baltimore Maryland USA

**Keywords:** cytokines, fever, heat shock proteins, innate immunity, tuberculosis

## Abstract

Fever is a common clinical symptom in patients with tuberculosis (TB). During fever, heat‐shock proteins (HSPs), such as HSP70, are expressed, which are molecular chaperones regulating protein folding and may also have immunomodulatory properties. How fever modulates immune responses during TB and by which mechanisms is unknown. In this study, we investigated the effects of fever, and specifically the role of HSP70, on *Mycobacterium tuberculosis* (*Mtb)*‐induced macrophage inflammatory responses. Human monocyte‐derived macrophages (MDM) were infected with *Mtb* at 37°C or 40°C to mimic febrile conditions. Fever suppresses *Mtb*‐induced IL‐1β and IL‐10 gene expression and secretion from MDM, but enhances *Mtb*‐induced HSP70 secretion and intracellular accumulation in MDM. Extracellular HSP70 and HSP70‐expressing macrophages are abundant in granulomas in TB patient biopsies. HSP70 antagonism decreases *Mtb*‐induced IL‐1β secretion during febrile conditions but has no significant effect on IL‐10 secretion. Pretreatment of MDM with recombinant HSP70 significantly increases *Mtb*‐induced IL‐1β at 37°C. Finally, extracellular HSP70 negatively regulates further HSP70 secretion from MDM during *Mtb* infection. Overall, fever and subsequent HSP70 expression modulates proinflammatory innate immune response in TB, which may have implications for the development of host‐directed therapies.

## Graphical abstract

*Mycobacterium tuberculosis* infection of human monocyte‐derived macrophages during febrile conditions significantly induces heat shock protein‐70 (HSP70) expression, which modulates cytokine responses. This highlights that pathophysiological responses, such as fever, regulate tuberculosis immunopathology.

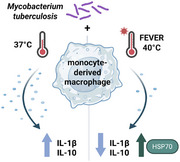

**Abbreviations**
HSP70: heat shock protein‐70
HSPs: heat shock proteins
MDM: monocyte‐derived macrophages
*Mtb*
: *Mycobacterium tuberculosis*
PBMCs: peripheral blood mononuclear cells
TB: tuberculosis

## 1 Introduction

Despite efforts to end the global tuberculosis (TB) epidemic by 2030 [1], 10.8 million people were infected with the causative agent, *Mycobacterium tuberculosis* (*Mtb*), and 1.25 million people died from TB in 2023 [1]. Furthermore, the annual number of TB deaths has risen due to the COVID pandemic [2]. *Mtb* typically causes pulmonary disease, characterized by tissue destruction and acute lung cavitation [3]. The rise of single‐, multi‐, and extensively drug‐resistant *Mtb* strains represents a major clinical challenge, despite the development of a few new antimycobacterial drugs [4].

Alveolar macrophages are one of the first responders to inhaled *Mtb* bacilli, and macrophages are a key immune cell during *Mtb* infection. Activated macrophages secrete proinflammatory cytokines and chemokines, including IL‐8, IL‐1β, and TNFα [5], and immunomodulatory mediators such as IL‐10. In addition, macrophages are a major source of matrix metalloproteinases (MMPs), a family of extracellular matrix (ECM) degrading enzymes that mediate tissue destruction and cavitary disease observed in TB [6].

Fever is defined as a core body temperature greater than 38°C and is a complex pathophysiological response to injury or infection [7, 8]. TB is one of the most common infectious diagnoses in patients presenting with fever of unknown origin, with 56% of patients with pulmonary or extra‐pulmonary TB presenting with fever [9]. During infection, fever can be triggered by exogenous pyrogens or pathogen‐associated molecular patterns (PAMPs) to drive production of pyrogenic cytokines, including IL‐1, TNFα, and IL‐6. It is unclear whether the induction of fever during infectious diseases, such as TB, is beneficial or detrimental to the host. Infection‐induced fever may be beneficial by activating innate immunity and the release of proinflammatory cytokines [7], and by creating less favorable conditions for replicating pathogens. Fever also enhances adaptive immunity by promoting T cell migration and differentiation into Th17 cells [10, 11]. The role of fever in TB and whether fever modulates innate immune responses during *Mtb* infection remains unknown.

A consequence of fever and cellular stress is the expression of heat shock proteins (HSPs), a highly conserved family of intracellular molecular chaperones that facilitate DNA repair [12]. HSP70, an ATP‐dependent molecular chaperone that stabilizes nascent or denatured proteins [13], is one of the most ubiquitous HSPs, with 17 genes and 30 pseudogenes identified in the human genome [14]. It is now apparent that HSPs have immunomodulatory functions [15], and HSPs have been identified as novel biomarkers in patients with TB. Patients with active pulmonary TB have elevated serum concentrations of HSP25, HSP60, HSP70, and HSP90 compared with controls [16]. Proteomic analysis of peripheral blood mononuclear cells (PBMCs) from patients with pulmonary TB showed downregulation of HSP70, HSP90, and HSP105 expression after 2 or 6 months of anti‐TB treatment [17].

Although HSPs are typically found intracellularly, HSP70 may be released from necrotic or damaged cells. Extracellular HSP70 can act as a damage‐associated molecular pattern (DAMP) to trigger proinflammatory responses [18]. Whether HSPs induced during febrile responses in TB modulate innate immune responses is ill‐defined. We hypothesized that fever regulates macrophage‐dependent cytokine responses via HSPs during *Mtb* infection. Therefore, we investigated the effects of fever‐range hyperthermia, and specifically the role of HSP70, on *Mtb*‐induced macrophage inflammatory responses in both a cellular model of *Mtb* infection and in TB patients.

## 2 Results

### 2.1 Fever Suppresses *Mtb*‐Induced IL‐1β and IL‐10 Secretion from Human MDM

**FIGURE 1:**
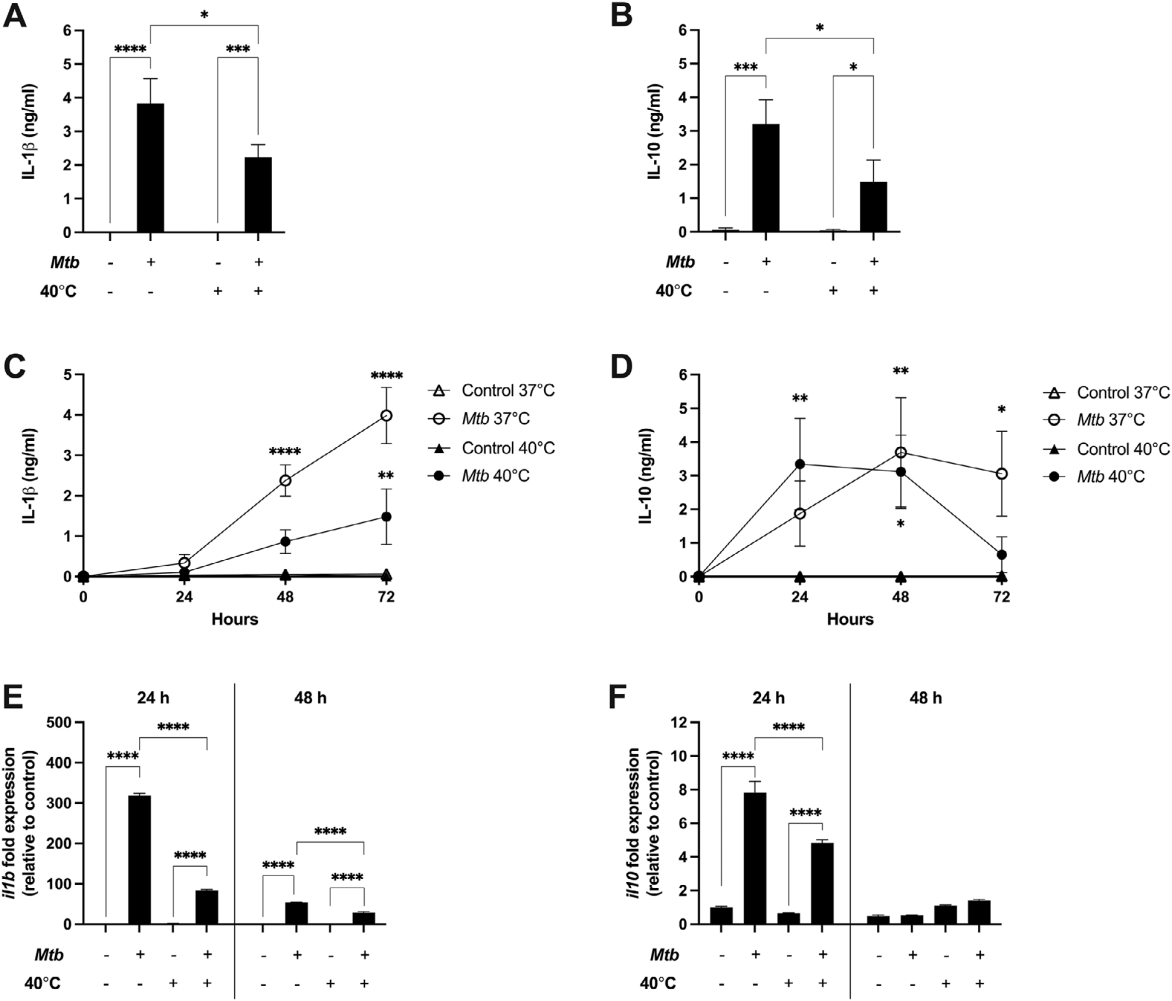
Fever suppresses *Mtb*‐induced IL‐1β and IL‐10 secretion and gene expression in human MDM. (A) IL‐1β and (B) IL‐10 secretion from *Mtb*‐stimulated or control MDM (*n* = 6–7 donors) incubated at 37°C or 40°C for 72 h. (C) IL‐1β and (D) IL‐10 secretion from *Mtb*‐stimulated (circles) or control (triangles) MDM (*n* = 3 donors) incubated at 37°C (open) or 40°C (black) for *t* = 0, 24, 48, or 72 h. (E) *il1b* and (F) *il10* fold change in gene expression from *Mtb*‐stimulated or control MDM (*n* = 3 donors) at 37°C or 40°C for 24 or 48 h. Fold change in gene expression is relative to time‐matched 37°C control MDM. Mean ± SEM are shown. Two‐way ANOVA statistical testing was performed (*p < 0.05, **p < 0.01, ***p < 0.001, ****p < 0.0001). In (C) and (D), asterisks refer to comparisons between *Mtb*‐stimulated and control MDM, cultured at the same temperature.

We initially investigated the effect of fever on monocyte‐derived macrophage (MDM) responses in an established cellular model of *Mtb* infection. MDM were stimulated with *Mtb* for 72 h at either 37°C or 40°C to model body temperature during febrile conditions. Culture of MDM at 40°C did not affect cell viability compared with culture at 37°C (data not shown). However, stimulation of MDM with *Mtb* at 40°C led to significantly greater IL‐1β secretion (2.24 ± 0.98 ng/mL) compared with control cells, but significantly less IL‐1β secretion than from MDM stimulated with *Mtb* in afebrile conditions at 37°C (3.83 ± 1.94 ng/mL, *p* = 0.022, Figure 1A). Fever also significantly decreased *Mtb*‐induced IL‐10 secretion from MDM compared with infected MDM cultured at 37°C (*p* = 0.042, Figure 1B).

To further characterize the impact of fever on cytokine secretion from MDM, the kinetics of IL‐1β secretion induced by *Mtb* stimulation at 37°C or 40°C was investigated in a separate experiment. Fever significantly suppressed *Mtb*‐induced IL‐1β secretion from MDM after 48 and 72 h of *Mtb* stimulation compared with culture at 37°C (both *p* < 0.0001, Figure 1C). A different pattern was observed for IL‐10 secretion. Fever induced greater IL‐10 secretion in *Mtb*‐stimulated MDM at 24 h compared with infected MDM cultured at 37°C, although this did not reach statistical significance (Figure 1D). However, after 72 h of *Mtb* infection, fever significantly decreased IL‐10 secretion compared with MDM infected at 37°C (Figure 1D). These data demonstrate that fever does not affect cell viability, but significantly decreases proinflammatory IL‐1β and anti‐inflammatory IL‐10 secretion from *Mtb‐*stimulated human MDM.

### 2.2 Fever Suppresses *Mtb*‐Induced *il1b* and *il10* Gene Expression in MDM

Next, we investigated whether *Mtb‐*induced MDM cytokine gene expression was affected by fever. Culture at 40°C significantly reduced *Mtb‐*stimulated MDM *il1b* gene expression at both 24 and 48 h compared with *Mtb*‐infected MDM at 37°C (both *p* < 0.0001, Figure 1E). However, *Mtb* stimulation for 24 or 48 h at 40°C significantly upregulated *il1b* expression in MDM compared with control cells (both *p* < 0.0001, Figure 1E). *Mtb* stimulation for 24 h at 40°C led to reduced *il10* gene expression compared with stimulation at 37°C (*p* < 0.0001, Figure 1F). As expected, stimulation of MDM with *Mtb* at either temperature led to greater *il10* expression than in control cells (both *p* < 0.0001, Figure 1F). After 48 h of *Mtb* stimulation at either 37°C or 40°C, *il10* expression returned to baseline and was similar in both stimulated and control cells (Figure 1F). Thus, fever suppresses IL‐1β and IL‐10 gene expression in *Mtb*‐stimulated MDM.

Although fever did not affect host cell viability in our model, it could potentially modulate pathogen functions [7] to impact our findings. Therefore, we investigated the effect of fever on bacterial cell load in our model. The growth of *Mtb* in 7H9 broth at 40°C was significantly reduced compared with growth at 37°C (Figure S1A). However, MDM stimulated with *Mtb* for 72 h at either 37°C or 40°C had a similar bacterial load (Figure S1B). Therefore, fever does not alter the *Mtb* infection burden in human MDM despite inhibitory effects in broth culture.

### 2.3 HSP70 is Highly Expressed in Granulomatous TB Tissue

**FIGURE 2:**
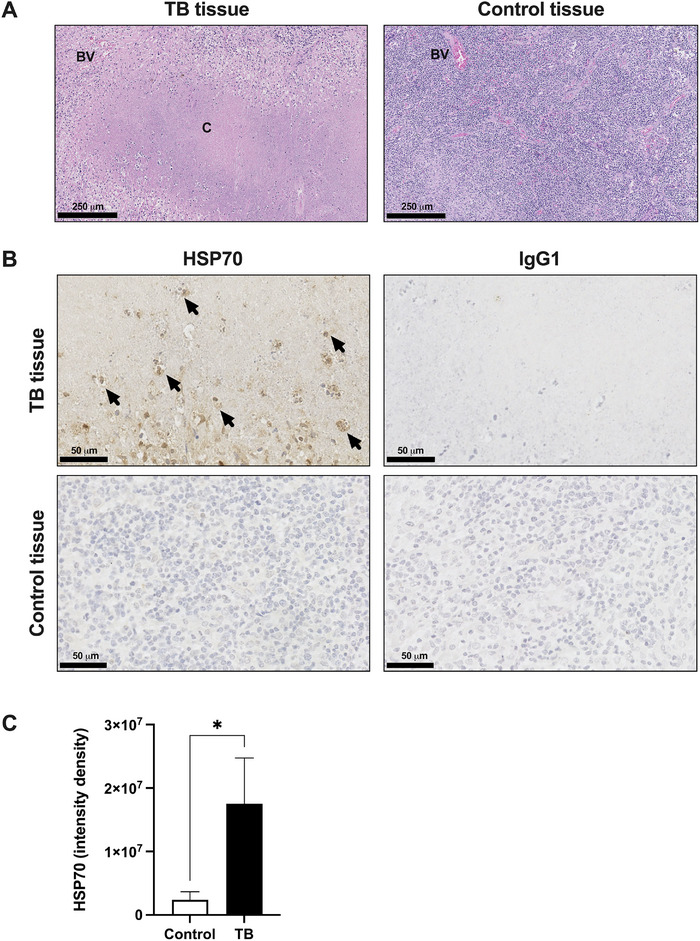
Intracellular and extracellular HSP70 are expressed in granulomatous TB tissue. (A) Representative histology images of TB‐positive (left) or TB‐negative control (right) patient lymph node tissue (*n* = 4). Sections stained with H&E were imaged at 10× as denoted by the 250 µm scale bar (BV = blood vessel, C = caseous center). (B) Representative immunohistochemical HSP70 (left) or IgG1 isotype control (right) stained TB‐positive (top row) or TB‐negative control (bottom row) patient lymph node tissue (*n* = 4). Images are shown at 40x magnification as indicated by the 50 µm scale bar. Black arrows denote HSP70^+^ macrophages or foam cells. (C) Quantification of HSP70 expression in TB‐positive or TB‐negative control patient lymph node tissue (*n* = 4). Mean ± SEM are shown. Mann–Whitney *U* test (*p < 0.05).

HSPs are induced during febrile conditions. Elevated plasma concentrations of HSPs have been found in TB patients compared with healthy controls [16], but the expression of HSP70 in TB patient tissue is unknown. We examined lymph node tissue biopsies from patients with TB lymphadenitis, which showed the presence of characteristic granulomas with central caseous necrosis (Figure 2A). Granulomas were not present in TB‐negative control tissue (Figure 2A). Both extracellular and intracellular HSP70 expression were readily detected in lymph node tissue from patients with TB lymphadenitis compared with isotype control‐stained sections (Figure 2B). Intracellular HSP70 was also found localized to macrophages or foam cells within granulomas (Figure 2B). Overall, there was significantly more HSP70 expression in TB‐positive tissue compared with TB‐negative controls (Figure 2B,C).

### 2.4 Febrile Conditions Induce HSP70 Secretion from *Mtb*‐Stimulated MDM

**FIGURE 3:**
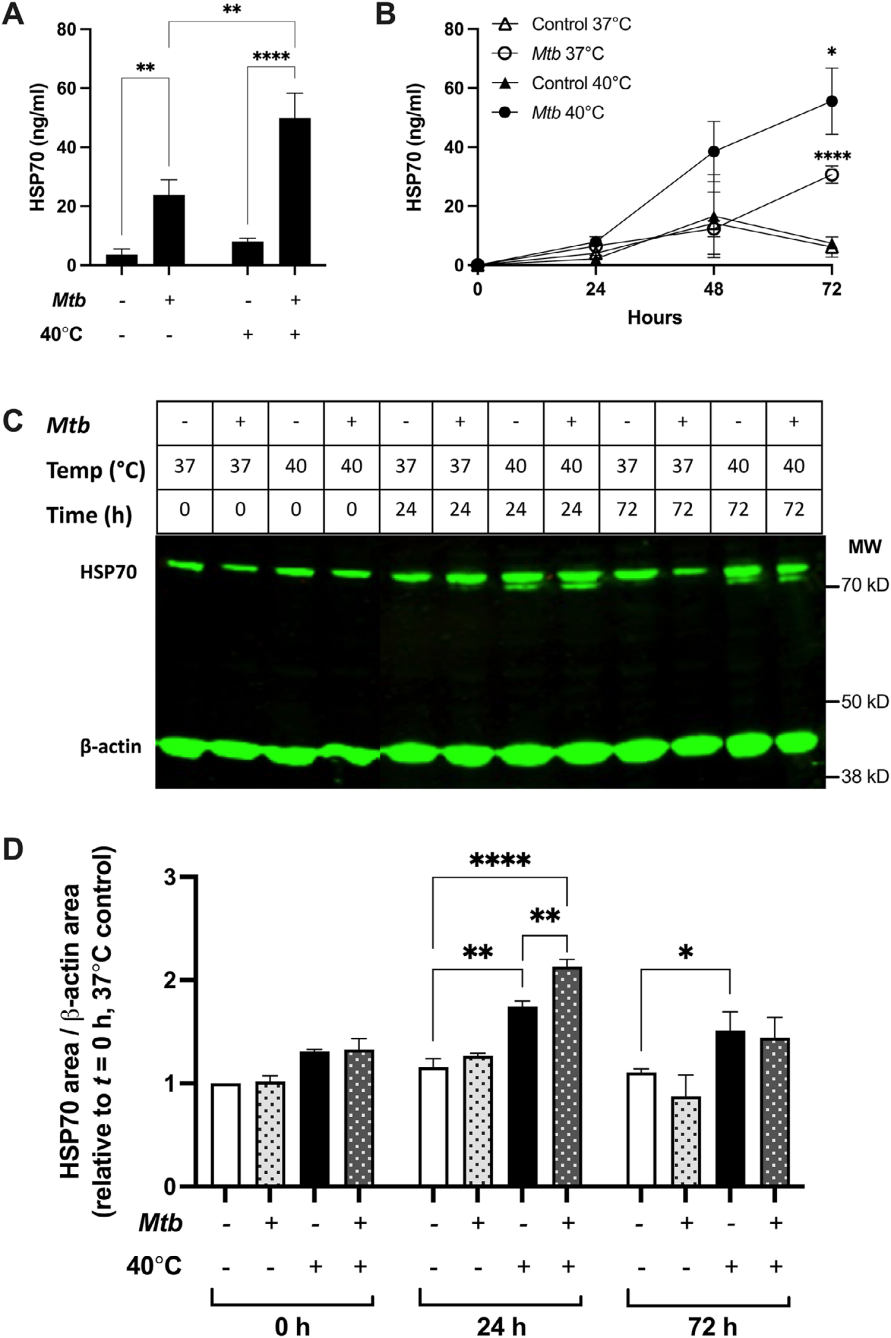
Fever increases HSP70 secretion from *Mtb*‐infected MDM and drives intracellular HSP70 accumulation. (A) HSP70 secretion from *Mtb*‐stimulated or control MDM (*n* = 6 donors) at 37°C or 40°C for 72 h. (B) Kinetics of HSP70 secretion from *Mtb*‐stimulated (circles) or control (triangles) MDM (*n* = 3 donors) incubated at 37°C (open) or 40°C (black) for 0, 24, 48, or 72 h. (C) Western blotting for intracellular HSP70 expression in *Mtb*‐stimulated or control MDM incubated at 37°C or 40°C for 0, 24, or 72 h. β‐actin was used as a loading control. Representative image showing one donor from three independent experiments. Original scans, as depicted in Figure S2, were cropped to only show the area of interest. (D) Quantification of HSP70 protein expression relative to β‐actin loading controls. Protein expression is normalized against *t* = 0 h, 37°C media control (*n* = 3 donors). Mean ± SEM are shown. Two‐way ANOVA statistical testing was performed (*p < 0.05, **p < 0.01, ****p < 0.0001). In Figure 4B, asterisks refer to comparisons between *Mtb*‐stimulated and control MDM cultured at the same corresponding temperature.

Since HSP70 was found in tissue from patients with TB, we next investigated whether *Mtb*‐infected human MDM express and secrete HSP70. At 72 h, MDM stimulated with *Mtb* at 40°C secreted significantly more HSP70 than *Mtb*‐stimulated MDM at 37°C (49.85 ± 20.67 vs. 23.93 ± 12.39 ng/mL, respectively, *p* = 0.003, Figure 3A). MDM stimulated with *Mtb* for 72 h at both 40°C and 37°C secreted significantly more HSP70 than control cells (*p* < 0.0001 and *p* < 0.01, respectively, Figure 3A). Since *Mtb*‐infected MDM secreted HSP70, we next explored further the kinetics of HSP70 secretion from MDM. Secreted HSP70 was detected after 48 h of *Mtb* stimulation at 40°C, and concentrations increased at 72 h (Figure 3B). Fever significantly enhanced *Mtb*‐induced HSP70 secretion compared with stimulated MDM cultured at 37°C after 48 h (38.52 ± 17.67 vs. 12.33 ± 4.752 ng/ml, *p* = 0.015, Figure 3B) or 72 h (55.58 ± 19.39 vs. 30.67 ± 5.13 ng/mL, *p* = 0.021, Figure 3B). Intracellular HSP70 expression was also assessed in MDM lysates after *Mtb* stimulation at 37°C or 40°C (Figure 3C). Culture of MDM in febrile conditions for 24 or 72 h significantly increased intracellular HSP70 expression compared with time‐matched control MDM cultured at 37°C (Figure 3C,D). *Mtb* stimulation enhanced fever‐induced intracellular HSP70 expression at 24 h, but this was not sustained at 72 h (Figure 3C,D). These data demonstrate that both *Mtb* stimulation and fever induce HSP70 secretion from MDM, but only fever increases intracellular HSP70 expression.

### 2.5 HSP70 Antagonism Decreases *Mtb*‐Induced IL‐1β and IL‐10 Secretion and Gene Expression in MDM

**FIGURE 4:**
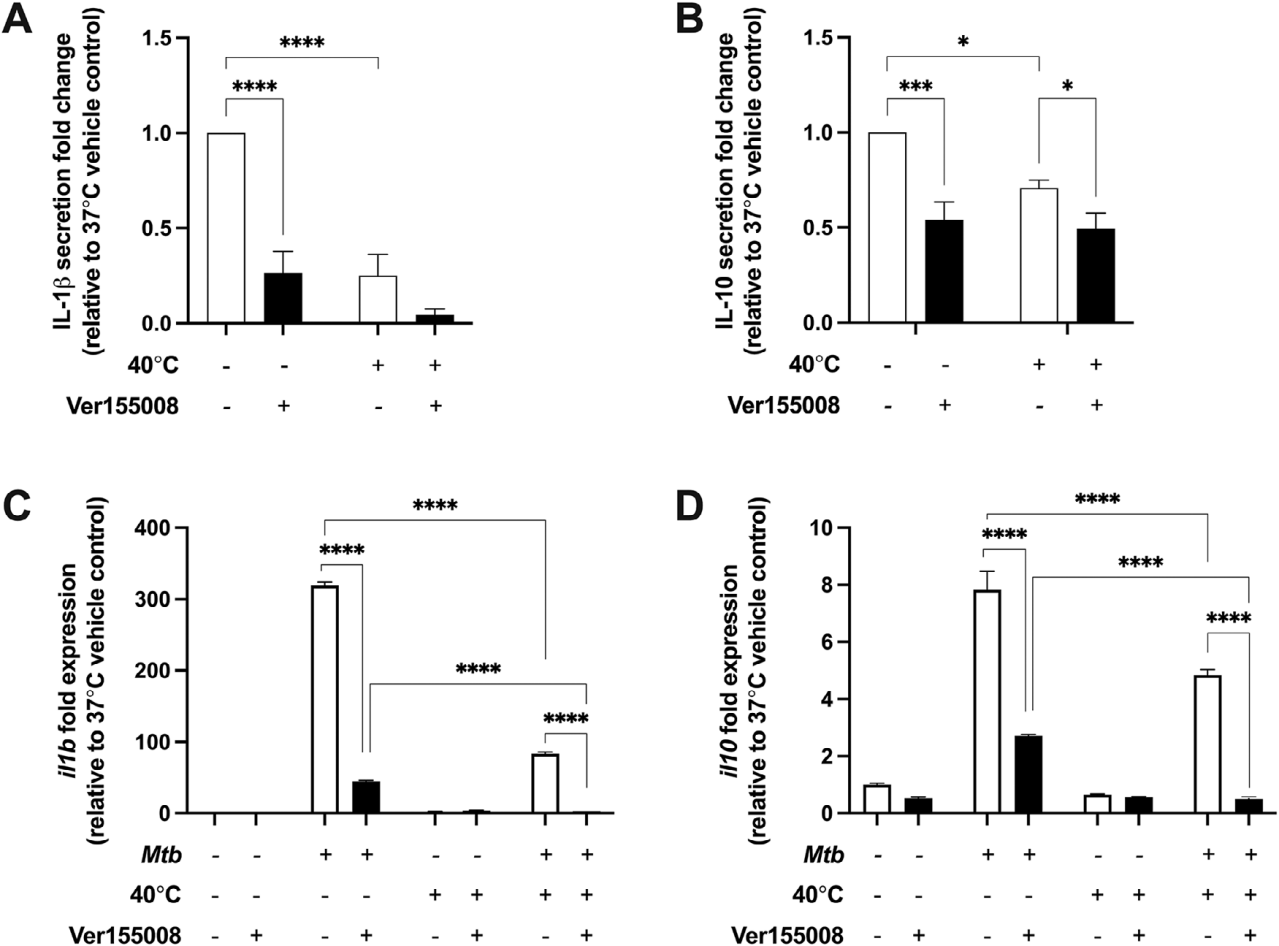
HSP70 antagonism decreases *Mtb*‐stimulated IL‐1β and IL‐10 secretion and gene expression from MDM at 37°C and 40°C. (A) IL‐1β and (B) IL‐10 secretion from *Mtb*‐stimulated MDM, either pretreated with vehicle (open bars) or with 25 µM HSP70 antagonist Ver155008 (black bars, *n* = 6 donors) at 37°C or 40°C for 24 h. Fold changes in secretion are relative to vehicle control pretreated *Mtb*‐infected MDM at 37°C. (C) *il1b* and (D) *il10* fold change in gene expression from *Mtb*‐stimulated MDM, either pretreated with vehicle (open bars) or with 25 µM Ver155008 (black bars, *n* = 3 donors) at 37°C or 40°C for 24 h. Fold change in gene expression is relative to the vehicle control pretreated control MDM at 37°C. Mean ± SEM are shown. Two‐way ANOVA statistical testing was performed (*p < 0.05, ***p < 0.001, ****p < 0.0001).

MDM were next pretreated with Ver155008, a HSP70 antagonist, prior to *Mtb* stimulation for 24 h to investigate the role of HSP70 in modulating cytokine responses in MDM. HSP70 antagonism with Ver155008 did not affect MDM cell viability (Figure S3). However, Ver155008 pretreatment did significantly decrease *Mtb*‐induced IL‐1β secretion (108.6 ± 25.4 pg/mL) compared with vehicle‐treated MDM at 37°C (333.3 ± 52.6 pg/mL, *p* < 0.0001), with no effect at 40°C (*p* = 0.08, Figure 4A). As expected, vehicle pretreated cells secreted less IL‐1β after *Mtb* stimulation at 40°C (114.1 ± 33.3 pg/mL) than at 37°C (333.3 ± 52.6 pg/mL, *p* < 0.0001, Figure 4A). Ver155008 pretreatment of *Mtb*‐stimulated MDM significantly reduced IL‐10 secretion compared with vehicle‐treated MDM at both 37°C (1.58 ± 0.19 vs. 3.97 ± 0.51 ng/mL, *p* < 0.01) or 40°C (1.55 ± 0.17 vs. 2.93 ± 0.40 ng/mL, *p* < 0.01, Figure 4B). However, Ver155008 pretreatment of MDM did not affect *Mtb*‐stimulated IL‐10 secretion at 40°C compared with stimulation at 37°C (*p* = 0.634, Figure 4B).

Ver155008 pretreatment of *Mtb*‐stimulated MDM significantly decreased *il1b* gene expression compared with *Mtb*‐stimulated vehicle‐treated MDM at both 37°C and 40°C (both *p* < 0.0001, Figure 4C). Similarly, Ver155008 pretreatment significantly decreased *Mtb*‐induced *il10* gene expression at 37°C and 40°C compared with *Mtb*‐stimulated vehicle‐treated MDM (Figure 4D). Such a reduction induced by Ver155008 pretreatment was additive to the decrease in *il1b* and *il10 gene* expression driven by *Mtb* stimulation at 40°C compared with expression at 37°C (both *p* < 0.0001, Figures 4C,D). In summary, HSP70 antagonism significantly decreases *Mtb*‐induced secretion and gene expression of IL‐1β and IL‐10, showing that extracellular HSP70 is key in driving these early immune responses in *Mtb*‐stimulated MDM.

### 2.6 HSP70 Induces IL‐1β Secretion from Infected MDM and Regulates Its Own Secretion by a Feedback Loop

**FIGURE 5:**
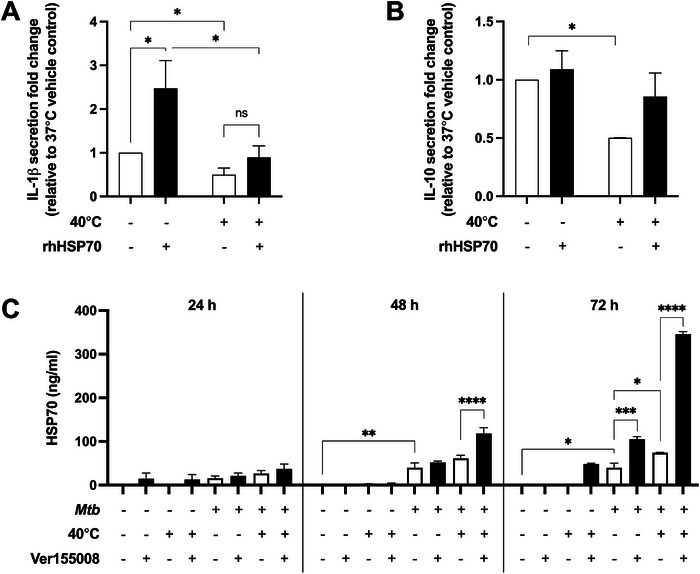
Recombinant HSP70 increases IL‐1β secretion at 37°C, and inhibition of HSP70 activity increases *Mtb*‐induced HSP70 secretion at 37°C, and to a greater extent at 40°C. (A) IL‐1β and (B) IL‐10 secretion from *Mtb*‐stimulated MDM, either pretreated with vehicle (open bars) or with 500 ng/mL recombinant human HSP70 (rhHSP70, black bars, *n* = 4–6 donors) at 37°C or 40°C for 24 h. Fold changes in secretion are relative to vehicle control pretreated *Mtb*‐infected MDM at 37°C. (C) HSP70 secretion from *Mtb*‐stimulated MDM, either pretreated with vehicle (open bars) or with 25 µM Ver155008 (black bars, *n* = 4 donors) at 37°C or 40°C for 24, 48, or 72 h. Mean ± SEM are shown. Two‐way ANOVA statistical testing was performed (*p < 0.05, **p < 0.01, ***p < 0.001, ****p < 0.0001).

As HSP70 antagonism significantly reduced IL‐1β and IL‐10 expression in *Mtb*‐stimulated MDM, the effects of exogenous HSP70 on MDM was examined. Pretreatment of MDM with recombinant human HSP70 (rhHSP70) prior to *Mtb* stimulation significantly increased IL‐1β secretion (702.0 ± 58.3 pg/mL) compared with stimulated vehicle controls at 37°C (280.7 ± 27.9 pg/ml, *p* = 0.018, Figure 5A), with no effect at 40°C (313.4 ± 25.7 vs. 251.9 ± 60.0 pg/mL, *p* = 0.435, Figure 5A). Pretreatment of MDM with rhHSP70 had no significant effect on IL‐10 secretion compared with vehicle controls at 37°C (3.32 ± 0.76 vs. 3.00 ± 0.78 ng/mL, *p* = 0.63) or 40°C (1.83 ± 0.86 vs. 1.24 ± 0.36 ng/mL, *p* = 0.17, Figure 5B). Thus, extracellular rhHSP70 drives *Mtb*‐induced secretion of IL‐1β, but not IL‐10, at 37°C. The addition of rhHSP70 did not affect MDM cell viability (Figure S3).

The addition of rhHSP70 only partially enhanced cytokine secretion from *Mtb*‐stimulated MDM, so finally, we investigated HSP70 secretion from Ver155008 pretreated MDM. At 72 h, HSP70 antagonism by Ver155008 significantly increased *Mtb*‐induced HSP70 secretion from human MDM at 37°C compared with *Mtb*‐stimulated vehicle‐treated cells (*p* < 0.001, Figure 5C). This effect was greatly enhanced when Ver155008‐pretreated MDM were stimulated with *Mtb* at 40°C for 48 or 72 h (both *p* < 0.0001, Figure 5C). These data show that extracellular HSP70 negatively regulates further HSP70 secretion from MDM during *Mtb* infection.

## 3 Discussion

We show for the first time that fever‐range hyperthermia suppresses *Mtb*‐induced IL‐1β gene expression and secretion from human MDM compared with stimulation at 37°C. These fever‐induced responses were observed between 24 and 72 h post infection, with no short‐term effects of fever on cytokine responses being observed before 24 h of *Mtb* infection (data not shown). Macrophages elicit potent proinflammatory responses during infection with both lab and clinical *Mtb* isolates [19]. The importance of IL‐1 secretion in controlling early *Mtb* infection is highlighted in animal studies, where *Mtb*‐infected IL‐1 knockout mice have more granulomatous lesions than infected wild‐type mice [20]. However, excessive secretion of IL‐1β and other proinflammatory cytokines may worsen TB immunopathology [21]. Our findings show that although fever does not affect control of *Mtb* burden within host cells, fever may act to limit excessive innate proinflammatory responses, which may prevent chronic inflammatory tissue damage during *Mtb* infection.

In addition, we show that fever reduces *Mtb*‐induced IL‐10 secretion and gene expression in human MDM compared with stimulation at 37°C. These data are consistent with studies showing that LPS stimulation of PBMCs at 40°C significantly reduces IL‐10 secretion compared with stimulation at 37°C [22]. Macrophages express IL‐10 during *Mtb* infection, and blocking IL‐10 in MDM infected with H37Ra, an avirulent lab strain of *Mtb*, has been shown to promote *Mtb* intracellular survival in phagosomes [23]. However, in the context of our model using a virulent *Mtb* strain (H37Rv), a moderate reduction in IL‐10 expression by fever did not affect the amount of *Mtb* recovered from infected MDM. This indicates that other cellular functions are relatively unaffected. IL‐10 is a key immunomodulatory regulator, and IL‐10‐deficient mice have decreased *Mtb* bacterial load due to enhanced Th1 responses [24]. Therefore, it is conceivable that fever may control *Mtb* growth by the reduction of IL‐10 expression to enhance adaptive immune responses.

Fever induces expression of HSPs [25] to stabilize protein refolding during physiological stress. We show for the first time that fever greatly enhances the secretion of extracellular HSP70 from MDM in response to *Mtb* stimulation. Additionally, fever promotes intracellular HSP70 accumulation in MDM, although we did not identify which specific HSP70 isoforms were increased. Our data is consistent with other published studies. For example, Zhou et al. [26] showed that human monocytes subjected to heat shock at 42°C upregulate intracellular HSP70 expression. Furthermore, alveolar macrophages stimulated with heat‐killed *Mtb* also increase intracellular HSP70 expression [27].

In this study, we made the novel observation of increased intracellular and extracellular HSP70 expression in granulomatous tissue from patients with TB. This finding in clinical samples highlights the relevance of HSP70 in patients with TB and is consistent with our cellular observations that *Mtb* infection induces HSP70 secretion from macrophages. Whilst tissue‐specific expression of HSP70 at the site of TB disease has not been reported, other studies have shown that patients with active pulmonary TB have elevated serum concentrations of HSP70 compared with controls [16]. Additionally, alveolar macrophages from bronchoalveolar lavage from patients with TB expressed more HSP70 than controls [27].

HSP70 antagonism reduces *Mtb*‐induced IL‐1β, but not IL‐10, gene expression and secretion from MDM during febrile conditions, whereas the addition of exogenous HSP70 during *Mtb* stimulation has the opposite effect and increases IL‐1β gene expression and secretion. HSP70 expressed during febrile conditions may therefore act to reverse fever‐induced suppression of cytokine responses in *Mtb*‐infected macrophages. Similarly, human monocytes treated with HSP70 enhance the secretion of other proinflammatory cytokines, such as TNFα [28]. In addition, extracellular HSP70 can activate TLR2/TLR4/CD14‐ [28] and MyD88‐dependent signaling pathways [29], which may drive cytokine gene expression. Interestingly, in our model, HSP70 antagonism by Ver155008 stimulates more extracellular HSP70 secretion from MDM. This suggests that HSP70 may negatively regulate its own expression. HSP70 has been shown to negatively regulate its own expression via heat shock factor‐1 (HSF1) in yeast cells [30] or by binding to its own mRNA in HeLa cells [31].

In summary, the data presented in this study show that fever‐range hyperthermia modulates innate immune responses during *Mtb* infection by suppressing IL‐1β and IL‐10 cytokine responses, while HSP70 counteracts this immunosuppressive response. Furthermore, extracellular HSP70 regulates its own expression in macrophages. A better understanding of the immune‐pathophysiological responses in TB, including fever, will likely impact the responses to host‐directed immune or anti‐inflammatory therapies aimed at decreasing pathology and improving TB patient outcomes.

### 3.1 Data Limitations and Perspectives

This study has some limitations. First, expression of HSP70 was examined in lymph node tissue from patients with TB lymphadenitis and not in pulmonary tissue. Lung biopsies are extraordinarily infrequently required in TB and should not be taken for purely experimental purposes. Whilst the TB disease pathology and induction of innate immune responses are similar in both tissues, HSP70 expression may be localized to specific compartments within the lung that we have not addressed in this study. Additionally, we cannot comment on the amount of HSP70 in patient clinical samples, such as plasma or bronchoalveolar lavage, but this remains the focus of future investigations. Lastly, although we have established that HSP70 modulates gene expression of cytokines in macrophages, detailed mechanistic studies are required to gain a better understanding of how HSP70 regulates these cytokine responses.

Our research highlights for the first time that pathophysiological responses, such as fever, and subsequent expression of HSP70, impact the host response to *Mtb* infection. This better understanding of TB disease mechanisms may have implications for identifying novel host‐directed therapies to limit disease pathology. Finally, findings from this study may also be relevant to many other infectious diseases, where fever is a common clinical symptom, such as *Streptococcus pneumoniae* infection, where innate immune responses are also key in controlling the disease.

## 4 Materials and Methods

### 4.1 *Mtb* Culture

*Mtb* H37Rv (ATCC 27294 from the American Type Culture Collection, USA) was routinely cultured as previously described [32]. For infection experiments, H37Rv was used at the mid‐logarithmic growth phase as defined by an optical density reading of 0.6 at 600 nm. To determine colony‐forming units (CFU) counts, infected or control MDM were washed and lysates plated in triplicate on Middlebrook 7H10 agar (BD Biosciences) plates and left to grow for 2.5 weeks at 37°C.

### 4.2 Monocyte‐Derived Macrophage Cell Culture

Monocytes were isolated from single donor leukocyte cones (National Blood Transfusion Service, UK, NHS REC:17/WS/0249) by density centrifugation and adhesion purification as previously described [32]. Monocytes were counted using Trypan Blue live/dead cell exclusion and seeded at 5 × 10^5^ cell/well in 12‐well plates before differentiation into MDM in Roswell Park Memorial Institute (RPMI)‐1640 medium supplemented with 10% Foetal Calf Serum (FCS), 2 mM L‐Glutamine, 10 µg/mL ampicillin and 100 ng/mL M‐CSF (all from Thermo Fisher Scientific, UK) for 4 days at 37°C, 5% CO_2._ The next day, the media was changed to RPMI supplemented with 10% FCS, 2 mM L‐glutamine, and 10 µg/mL ampicillin, and MDM were cultured overnight at 37°C with 5% CO_2_. Cell viability was assessed by CyQUANT LDH cytotoxicity assay (Thermo Fisher Scientific).

### 4.3 MDM Infection

On the day of *Mtb* infection, RPMI‐1640 from MDM cultures was replaced with macrophage serum‐free media (M‐SFM) (Thermo Fisher Scientific). For H37Rv infection experiments, a multiplicity of infection (MOI) of 1 was used, and MDM were stimulated at 37°C or 40°C, 5% CO_2_ for 24, 48, or 72 h. As a control group, MDM were stimulated with 7H9 broth. In later experiments, MDM were pretreated with 25 µM Ver155008, a HSP70 antagonist (Santa Cruz Biotechnology, USA), or 500 ng/mL recombinant human HSP70 (rhHSP70, Bio‐Techne, UK) 1 h before infection.

### 4.4 ELISA

The concentration of secreted human IL‐1β, IL‐10, or HSP70 was quantified using sandwich ELISA Duoset assays (Bio‐Techne) according to the manufacturer's guidelines.

### 4.5 Quantitative Real‐Time PCR

Cell lysates for quantitative real‐time PCR (qRT‐PCR) were collected in TRIzol (Thermo Fisher Scientific) and total RNA was extracted and reverse‐transcribed into cDNA as previously described [32]. qRT‐PCR using Brilliant II master‐mix (Agilent, USA), TaqMan assays for *il10* (Hs00961622_m1), *il1b* (Hs01555410_m1) and *18s* (4310893E) (all from Thermo Fisher Scientific) were run on a CFX Connect real‐time PCR detection system (Bio‐Rad, UK) with the following thermal profile: 95°C for 10 min, followed by 40 cycles of 95°C for 30 s and 60°C for 1 min. Quantification of *il1b* or *il10* gene expression was normalized against the *18s* reference gene, and fold change in expression was calculated using the ΔΔCT method [33].

### 4.6 Immunohistochemistry

Immunohistochemistry was performed for the presence of HSP70 in paraffin‐embedded lymph node biopsies from four adult HIV‐negative, TB‐negative individuals and four HIV‐negative patients diagnosed with TB lymphadenitis (as confirmed by three independent histopathologists). The presence of acid‐fast Bacilli (AFB) in TB lymph node biopsies was also confirmed by histopathologists. Research ethical approval for patient recruitment was granted, and informed consent was obtained from all study participants. In brief, 5 µm sections were dewaxed before heat‐induced epitope retrieval in 10 mM Tris‐HCl (pH 10). Sections were permeabilized in Tris‐buffered saline (TBS)/0.2% Triton‐X100 for 5 min before blocking in TBS/10% goat serum/1% BSA for 1 h. Primary anti‐HSP70 antibody (10 µg/mL, cat no. ADI‐SPA‐812, Enzo BioChem, USA) or rabbit IgG isotype control (10 µg/mL, clone EPR25A, Abcam, UK) was incubated on sections overnight at 4°C. Sections were washed and endogenous peroxidase activity was blocked in 0.3% H_2_O_2_/TBS for 15 min. Goat anti‐rabbit IgG biotinylated secondary antibody was added to sections for 30 min before incubation with streptavidin‐peroxidase (both from Vector Laboratories, USA). Staining was developed with DAB (Vector Laboratories), followed by hematoxylin nuclear counter‐staining, dehydration, and mounting. Images were taken on a Nanozoomer digital slide scanner and analyzed using NDP.viewer software (both from Hamamatsu Corporation, Japan). Quantification of HSP70 staining was performed using Fuji analysis software [34].

### 4.7 Western Blot

Treated MDM were washed with PBS prior to cell lysate collection in SDS sample buffer [35] before boiling at 70°C for 10 min. Prepared lysates and Chameleon Duo prestained protein ladder (LI‐COR, USA) were run on NuPAGE 4–12% Bis‐Tris polyacrylamide gels (Thermo Fisher Scientific) at 200 V for 50 min. Separated proteins were transferred onto a nitrocellulose membrane (Thermo Fisher Scientific) at 30 V for 90 min. Blots were blocked in Odyssey blocking buffer (LI‐COR) diluted 1:1 in PBS for 1 h before incubation overnight at 4°C with HSP70 primary antibody (clone 5A5, Abcam, 1:1,000 dilution in blocking buffer) or β‐actin primary antibody (clone AC‐74, Sigma‐Aldrich, 1:10,000 dilution in blocking buffer). The next day, blots were washed three times before incubation with IRDye 800CW fluorescently conjugated goat anti‐mouse IgG secondary antibody (LI‐COR) for 30 min at RT. Blots were washed before imaging using an Odyssey DLx system (LI‐COR). Quantification of HSP70 expression relative to β‐actin loading control was performed using Fuji analysis software [34].

### 4.8 Statistical analysis

Experiments were conducted with at least three different biological donors, with experimental triplicates unless stated otherwise. All two‐way ANOVA statistical analyses with Holm–Sidak post hoc testing or Mann–Whitney *U*‐test were performed using Prism V10 (GraphPad, USA). Means ± SEM are shown, and *p* < 0.05 is considered statistically significant.

## Author Contributions

Jon S. Friedland conceptualized the study. Sajeel A. Shah and Jon S. Friedland were involved in experimental design. Deborah L. W. Chong, Sajeel A. Shah, Julia Kutschenreuter, Ramla Cusman, and Meena Murugananden Pillai performed and analyzed the experiments. Daniela E. Kirwan and Robert H. Gilman recruited and collected clinical samples. Deborah L. W. Chong and Jon S. Friedland wrote the manuscript. All authors read and approved the final manuscript.

## Ethical Approval Statement

Research ethical approval for patient recruitment, tissue collection, and usage for research was previously granted from the Universidad Peruana Cayetano Heredia, Lima (Peru) [36]. Informed consent was obtained from all study participants. Local ethical approvals were obtained to isolate and use MDM from leukocyte cones (NHS REC:17/WS/0249). Permission has been granted to publish data obtained from these studies using human cells or tissue.

## Conflicts of Interest

The authors declare no conflicts of interest.

## Supporting information




**Supporting File 1**: eji6019‐sup‐0001‐figuresS1‐S3.docx

## Data Availability

The data that support the findings of this study are available from the corresponding author upon reasonable request.
