# Supplementary material for: Fever‐Induced Heat Shock Protein‐70 Regulates Macrophage IL‐1β and IL‐10 Secretion During Mycobacterium tuberculosis Infection
Source: Eur J Immunol. 2025 Jul 16;55(7):e51963. doi: 10.1002/eji.202551963 (PMC12265389; doi:10.1002/eji.202551963)
Supplement: Supplementary file 1 — Supporting File 1: eji6019‐sup‐0001‐figuresS1‐S3.docx [file EJI-55-e51963-s001.docx]

**SUPPLEMENTAL FIGURES**

**
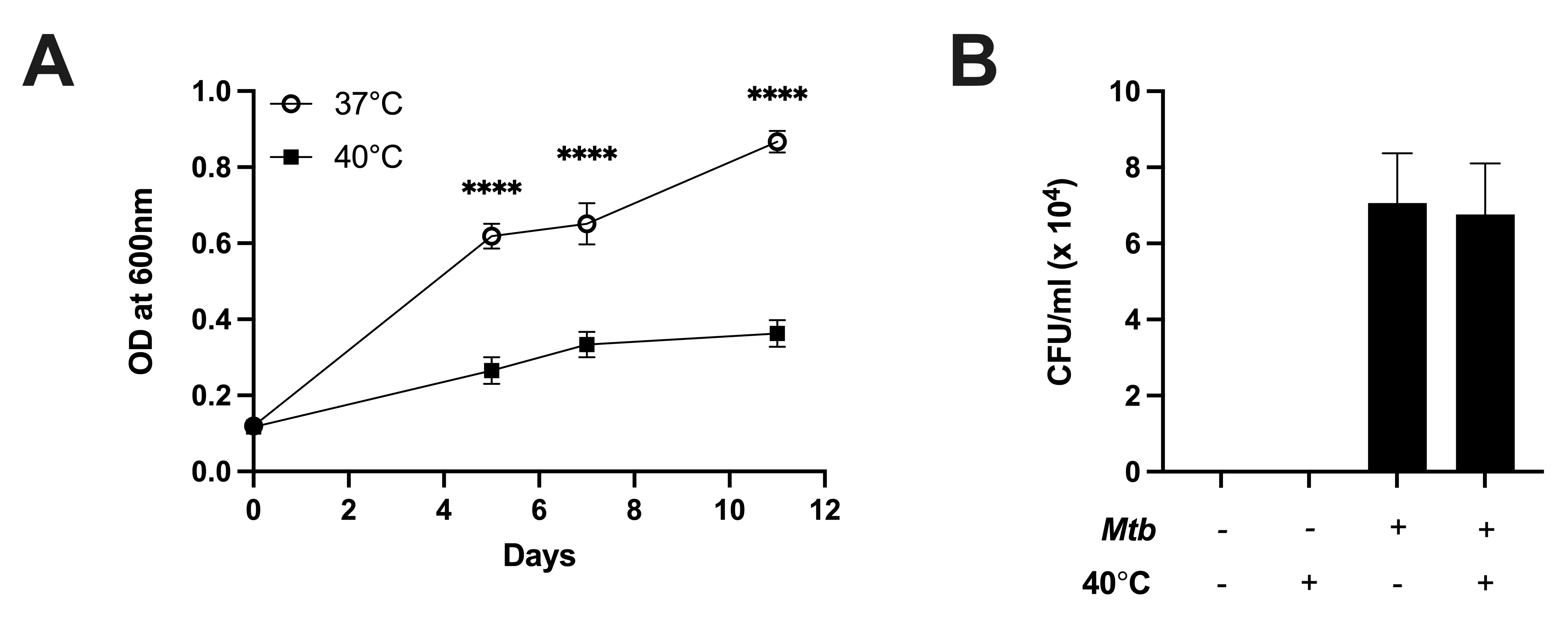
**

**Supplemental Figure 1. Fever reduces *Mtb* growth in broth, but does not affect infection burden in MDM.**

(A) *Mtb* was cultured in 7H9 broth at 37°C (open circles) or 40°C (black squares) for 11 days. Optical density (OD) at 600 nm was used to monitor growth over time. Representative data of three independent experiments. Two-way ANOVA statistical testing was performed (*****p*<0.0001). (B) *Mtb*-stimulated or control MDM were incubated at 37°C or 40°C (*n* = 5 donors). After 72 h, cells were washed and lysates plated on to 7H11 agar. Colony forming units (CFU) were calculated after 2.5 weeks growth.

**
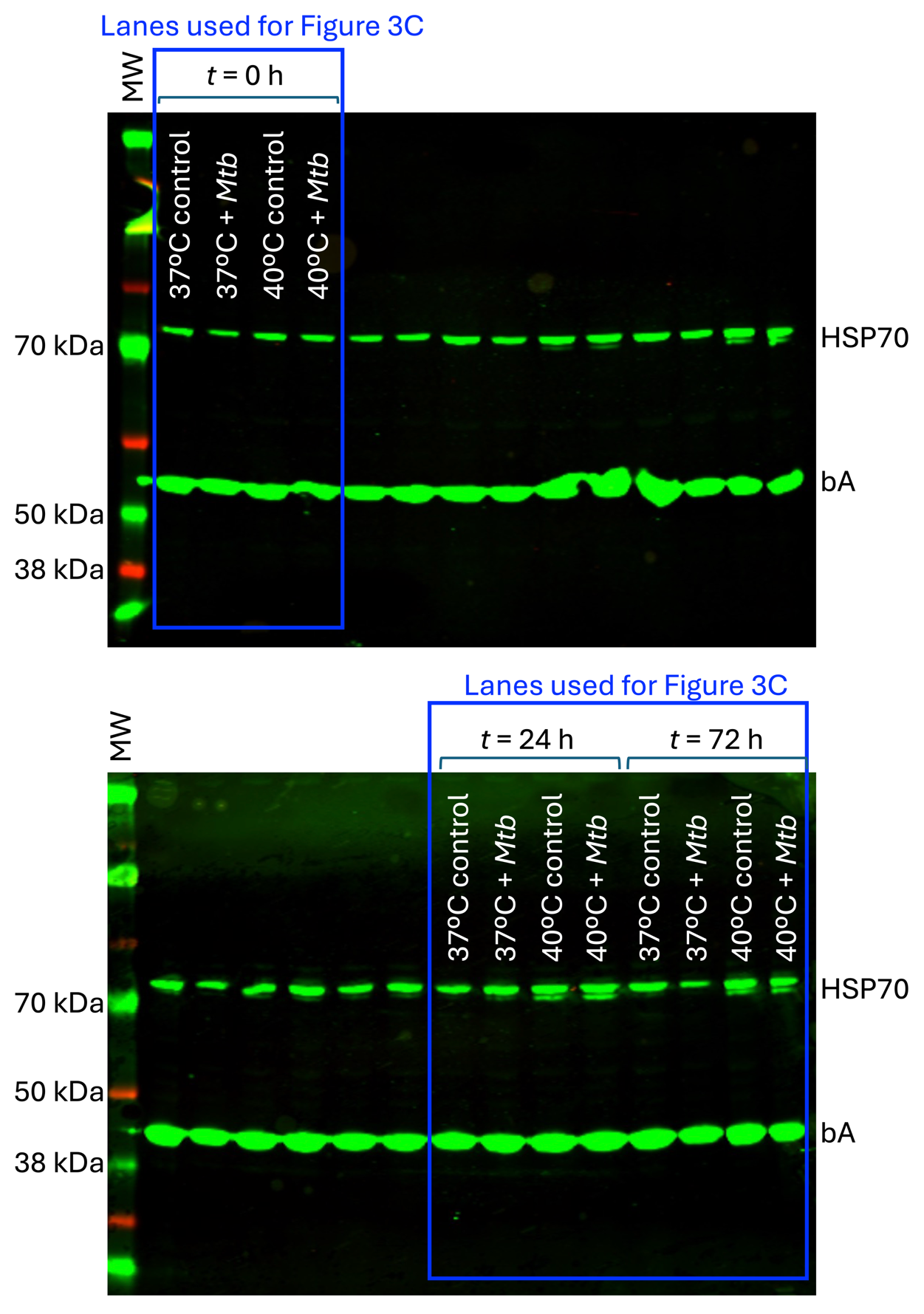
**

**Supplemental Figure 2. Raw Western blot image of HSP70 expression in *Mtb*-stimulated or control MDM.**

Representative Western blotting for intracellular HSP70 expression in *Mtb*-stimulated or control MDM incubated at 37°C or 40°C for 0, 24 or 72 h. β-actin (bA) was used as loading control. Image shown is from one MDM donor. Blue boxes denote lanes cropped together to form Figure 3C. Other lanes in the Western blot are irrelevant time-points in the stimulation experiment.

**
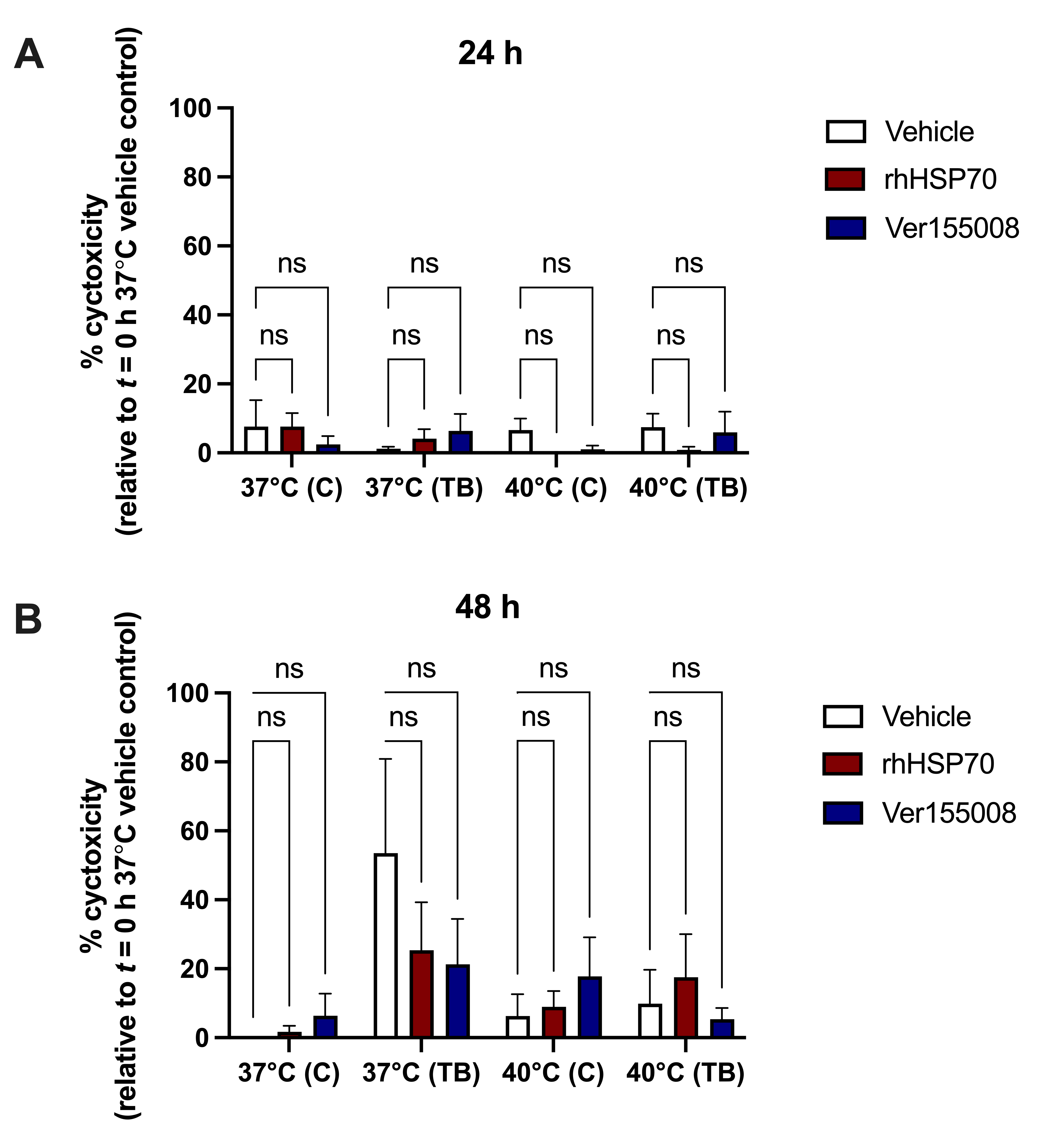
**

**Supplemental Figure 3. Stimulation of MDM with recombinant HSP70 or HSP70 antagonist does not affect cell viability.**

Cellular cytotoxicity was determined in *Mtb*-stimulated (TB) or control (C) MDM (*n* = 3 donors), pre-treated with vehicle control (white bars), 500 ng/ml recombinant human HSP70 (rhHSP70, red bars) or 25 μM Ver155008 (blue bars) at 37°C or 40°C for (A) 24 h or (B) 48 h. Percentage cytotoxicity is relative to *t* = 0 h vehicle pre-treated control MDM at 37°C. Two-ANOVA statistical testing was performed (ns = not significant).
